# Functional insights into modulation of BK_Ca_ channel activity to alter myometrial contractility

**DOI:** 10.3389/fphys.2014.00289

**Published:** 2014-07-31

**Authors:** Ramón A. Lorca, Monali Prabagaran, Sarah K. England

**Affiliations:** Department of Obstetrics and Gynecology, Washington University in St. Louis School of MedicineSt. Louis, MO, USA

**Keywords:** BK_Ca_ channel, ion channel modulation, myometrium, pregnancy, uterine contraction

## Abstract

The large-conductance voltage- and Ca^2+^-activated K^+^ channel (BK_Ca_) is an important regulator of membrane excitability in a wide variety of cells and tissues. In myometrial smooth muscle, activation of BK_Ca_ plays essential roles in buffering contractility to maintain uterine quiescence during pregnancy and in the transition to a more contractile state at the onset of labor. Multiple mechanisms of modulation have been described to alter BK_Ca_ channel activity, expression, and cellular localization. In the myometrium, BK_Ca_ is regulated by alternative splicing, protein targeting to the plasma membrane, compartmentation in membrane microdomains, and posttranslational modifications. In addition, interaction with auxiliary proteins (i.e., β1- and β2-subunits), association with G-protein coupled receptor signaling pathways, such as those activated by adrenergic and oxytocin receptors, and hormonal regulation provide further mechanisms of variable modulation of BK_Ca_ channel function in myometrial smooth muscle. Here, we provide an overview of these mechanisms of BK_Ca_ channel modulation and provide a context for them in relation to myometrial function.

## BK_Ca_ channel function in myometrium

The myometrium, the middle layer of the uterine wall responsible for uterine contractions, undergoes marked structural and functional modifications throughout pregnancy. During most of gestation, the myometrium remains in a quiescent state, whereas at the onset of labor, it becomes highly contractile to deliver the newborn. Regulation of myometrial contractility during pregnancy, and in particular labor, has been the focus of many studies, but the mechanisms controlling the transition from quiescence to contractility are intricate and remain elusive. Moreover, this transition is often mistimed; in the U.S., approximately 12% of babies are born prematurely and up to 10% of pregnancies are described as post-term (Gulmezoglu et al., 2012; Martin and Osterman, 2013). Thus, understanding how this transition is controlled is essential to ensure the health of mothers and newborns.

Uterine contraction is primarily mediated by rises in cytoplasmic Ca^2+^ concentration and activation of Ca^2+^-calmodulin/myosin light chain kinase pathways (Wray, 1993; Bru-Mercier et al., 2012). The mechanisms that elicit increases in intracellular Ca^2+^ levels and contraction in myometrial smooth muscle cells (MSMCs) include: (i) Ca^2+^ influx through voltage-gated Ca^2+^ channels, (ii) agonist (e.g., acetylcholine or ATP) binding to receptor-operated channels, and (iii) binding of agonists (e.g., oxytocin) to receptors that evoke Ca^2+^ release from intracellular stores (Inoue et al., 1992; Wray, 1993; Sanborn, 2000). Additionally, the onset of labor requires the MSMCs to switch from a hyperpolarized to a more depolarized state. This transition is controlled, in part, by a complex regulation of ion channel activity. Multiple types of ion channels are responsible for changes in the membrane potential in MSMCs (Sanborn, 2000; Shmygol et al., 2007a; Chan et al., 2014); potassium channels, in particular, play an important role in controlling membrane potential and attenuating excitation to maintain quiescence in pre-labor MSMCs.

Several lines of evidence indicate that the large-conductance voltage- and Ca^2+^-activated K^+^ channel (BK_Ca_) is a key regulator of myometrial membrane potential and the maintenance of uterine quiescence. First, the BK_Ca_ channel is one of the most abundant potassium channels in myometrial tissue (Tritthart et al., 1991; Perez et al., 1993; Chan et al., 2014). Second, early reports described an outward K^+^ current activated by Ca^2+^ influx in MSMCs (Vassort, 1975); pharmacological characterization later attributed this current to the BK_Ca_ channel (Anwer et al., 1993). Third, inhibition of BK_Ca_ depolarizes MSMCs and increases myometrial contractility in both rat and human tissue (Anwer et al., 1993). Fourth, activity of BK_Ca_ channels evokes a large efflux of K^+^ and repolarization of the membrane. Finally, enhancing BK_Ca_ channel opening has a potent relaxant effect on myometrium from different species (Khan et al., 1998; Choudhury et al., 2011; Xu et al., 2011).

It must be noted that some evidence argues against the importance of the BK_Ca_ channel. For example, mice lacking the BK_Ca_ channel gene, *mSlo1*, give birth to smaller pups and litters, although they reach term successfully (Meredith et al., 2004); however, compensatory mechanisms to systemic channel ablation have not been addressed. Additionally, a few studies have shown a minimal effect of BK_Ca_ channel blockers or openers on rodent and human myometrial contraction *in vitro* (Aaronson et al., 2006; Smith et al., 2007; Sadlonova et al., 2011). However, as we shall see below, this channel is modulated by multiple factors that are difficult to replicate *in vitro*.

The BK_Ca_ channel is formed by homo-tetramers of α-subunits; each subunit comprises seven conserved transmembrane domains (S0 through S6), an extracellular N terminus, and a large C-terminal domain (Wallner et al., 1996; Meera et al., 1997). The C-terminal domain encompasses four hydrophobic segments (S7–S10), two predicted regulators of K^+^ conductance domains (RCK1 and RCK2), and a Ca^2+^ sensor domain. The pore-forming α-subunit is frequently associated with various auxiliary subunits, β1–β4 or γ1–γ4 (Knaus et al., 1994b; Wallner et al., 1999; Behrens et al., 2000; Brenner et al., 2000; Uebele et al., 2000; Yan and Aldrich, 2012), which confers further functional diversity.

**Figure 1:**
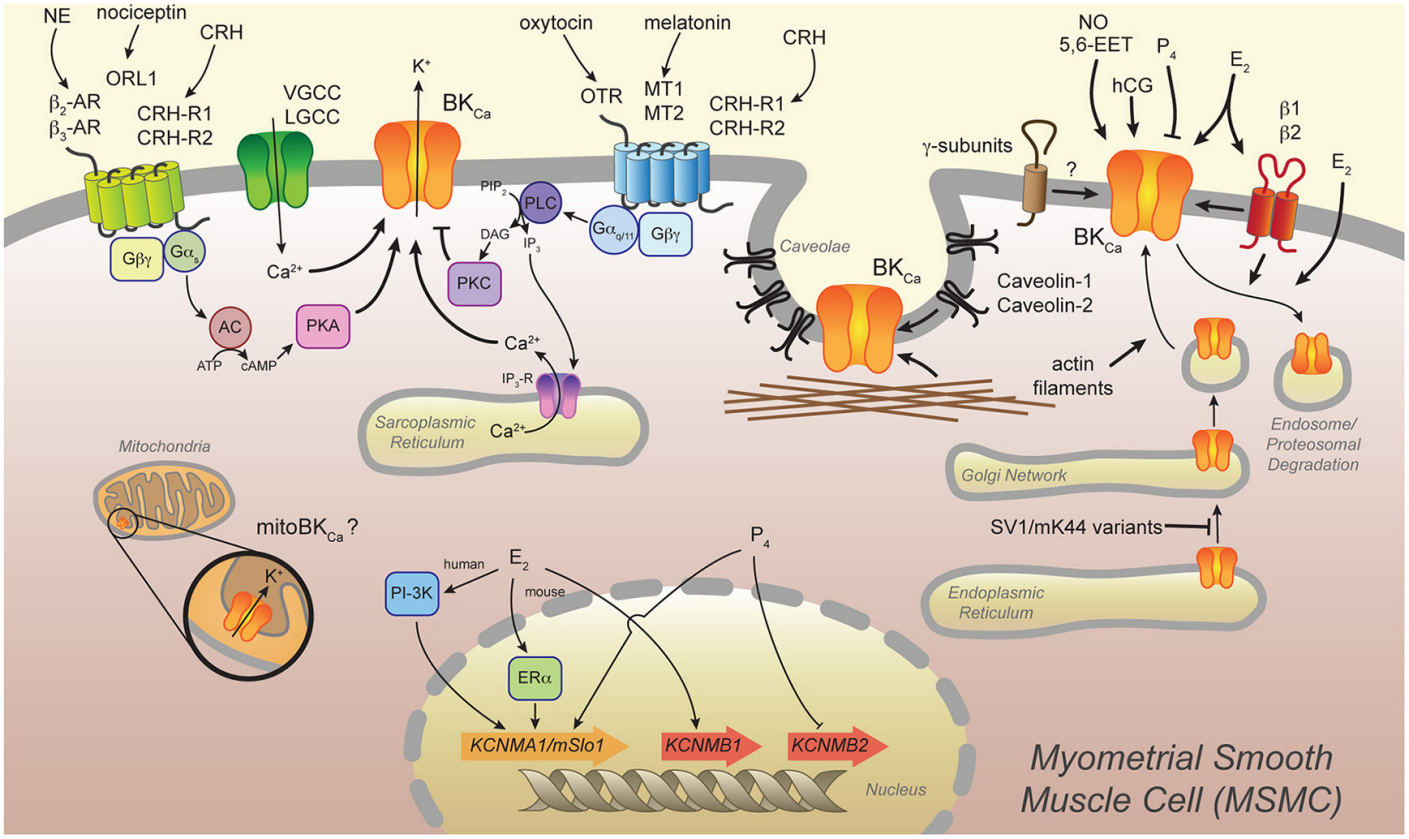
**Several mechanisms modulate the BK_Ca_ channel in the myometrium**. Certain splice variants (SV1 and mK44) of the BK_Ca_ channel are retained in the endoplasmic reticulum, whereas actin filaments induce traffic of BK_Ca_ to the plasma membrane of the myometrial smooth muscle cell (MSMC). Localization of BK_Ca_ channels in membrane microdomains (i.e., caveolae) and interaction with caveolin-1 and -2 and actin filaments modulate the channel's activity. The BK_Ca_ auxiliary β1- and β2-subunits modify channel activation by direct interaction and, in the case of β1, by inducing its internalization to endosomes. Novel BK_Ca_ auxiliary γ-subunits are expressed in the uterus, but their significance for MSMC excitability has not been assessed. The vasoactive molecules nitric oxide (NO) and epoxyeicosatrienoic acid (5,6-EET) induce relaxation of the myometrium likely by modulation of BK_Ca_ channel activity. The steroid hormones 17β-estradiol (E_2_) and progesterone (P_4_) are important in maintaining pregnancy and inducing labor. These hormones modulate activity of the BK_Ca_ channel in several ways: directly modulating BK_Ca_ channel activity, inducing proteosomal degradation of the channel, and regulating expression of the genes encoding the BK_Ca_ α-subunit (*KCNMA1*/*mSlo1*) or β-subunits (*KCNMB1* and *KCNMB2*). Another pregnancy-related hormone, human chorionic gonadotropin (hCG), modulates BK_Ca_ channel activity to induce relaxation of the myometrium. Several G-protein coupled receptors (GPCRs) regulate BK_Ca_ channel activity in MSMCs. Norepinephrine (NE) and nociceptin bind their receptors, β2- and β3-adrenoceptors (β2- and β3-AR) and the orphan opioid receptor-like 1 (ORL-1), respectively, and thereby activate G-proteins (Gα_s_, Gβγ). This leads to adenylyl cyclase (AC) production of cyclic AMP (cAMP), which activates protein kinase A (PKA) and modulates BK_Ca_ channel activity. Oxytocin and melatonin stimulate oxytocin receptor (OTR) and melatonin receptors 1 and 2 (MT1 and MT2), respectively, and thereby induce Gα_q/11_-dependent activation of phospholipase C (PLC). This leads to production of diacylglycerol (DAG), which in turn causes protein kinase C (PKC)-dependent phosphorylation of the BK_Ca_ channel. PLC also produces inositol 1,4,5-triphosphate (IP_3_) from membrane-bound phosphatidylinositol 4,5-bisphosphate (PIP_2_) and thereby brings about Ca^2+^ release from the sarcoplasmic reticulum. In addition to activation by Ca^2+^ release from intracellular stores, the BK_Ca_ channel is activated by Ca^2+^ influx from nearby voltage- or ligand-gated Ca^2+^ channels (VGCC and LGCC, respectively). Corticotropin-releasing hormone (CRH) binds to its receptors CRH-R1 and CRH-R2, which are linked to multiple signaling pathways and induce up- or down-regulation of BK_Ca_ channel activity. Finally, a particular BK_Ca_ channel (mitoBK_Ca_) targets to the inner membrane of mitochondria and may influence MSMC contractility.

Several mechanisms have been described to regulate BK_Ca_ channel function, such as expression of splice variants, compartmentation in membrane microdomains, posttranslational modifications, interaction with auxiliary proteins, and hormonal regulation. Here, we provide an overview of some of these mechanisms and discuss them in relation to myometrial function. Figure 1 provides a schematic representation of the mechanisms we describe.

## Intrinsic mechanisms of BK_Ca_ channel modulation

### Splice variants

The gene encoding the BK_Ca_ channel (*slo1*/*KCNMA1*) was first cloned from *Drosophila* (Atkinson et al., 1991; Adelman et al., 1992), and a mammalian gene was identified later (Butler et al., 1993). The BK_Ca_ channel is encoded by a single gene, and alternative splicing allows this channel to respond to a variety of regulatory inputs in a tissue-specific manner. To date, over 30 exons have been reported in the human *KCNMA1* gene (http://www.genecards.org/cgi-bin/carddisp.pl?gene=KCNMA1), leading to a large number of potential isoforms of the channel. Early studies demonstrated that splice variants of the BK_Ca_ channel have altered Ca^2+^ and voltage sensitivities (Tseng-Crank et al., 1994), and key phosphorylation sites are created by the inclusion of certain exons (Tian et al., 2001). In mouse myometrium, the expression of BK_Ca_ channel isoforms with low sensitivity to Ca^2+^ increases at mid-pregnancy (Benkusky et al., 2000). In human myometrium, expression of specific spliced isoforms can be altered during pregnancy and at the juncture between non-laboring and laboring states (Curley et al., 2004), allowing the uterus to attain a more excitable state during labor. For example, although the overall levels of BK_Ca_ channel transcript and protein decrease as term approaches (Matharoo-Ball et al., 2003; Gao et al., 2009), the proportion of the mK44 isoform transcript increases at this time (Curley et al., 2004). This isoform bears a unique 44 amino-acid insertion and undergoes endoproteolytic cleavage, with membrane localization of the N terminus variant and intracellular retention of the remaining cleaved pore-forming C terminus (Korovkina et al., 2006). Additionally, mK44 is less sensitive to Ca^2+^ and voltage than the canonical (lacking the insert) channel (Korovkina et al., 2001), suggesting that this isoform may modulate uterine activity near the time of labor (Curley et al., 2004).

Other splice variants that are widely expressed could play an important role in myometrial excitability during gestation, such as the stress axis regulated exon (STREX) isoform, which introduces 59 amino acids into the linker between cytosolic domains S8 and S9 (Saito et al., 1997). This idea is supported by studies showing that the STREX variant is regulated during pregnancy (Benkusky et al., 2000) in mice and rats by adrenocorticotropic hormone, estrogen, and progesterone (Xie and McCobb, 1998; Zhu et al., 2005). Additionally, STREX harbors a consensus PKA phosphorylation motif, whose phosphorylation inhibits channel activity (Tian et al., 2001). STREX expression decreases in rat myometrium during pregnancy, likely due to an estrogenic effect (Zhu et al., 2005) (see Section Hormonal regulation). Although this isoform does not appear to play a dominant role in human myometrium, it may affect myometrial excitability in other species.

Alternative splicing is usually considered a mechanism to derive variability from single gene products, but it may also regulate protein trafficking, as suggested by the existence of yet another splice variant termed SV1. In this protein, 33 amino acids that include an endoplasmic reticulum (ER) retention motif (CVLF) are inserted within the S1 transmembrane domain. Thus, this isoform is retained in the ER, where it acts as a naturally occurring dominant negative (Zarei et al., 2001). Although the role of this isoform in controlling myometrial excitability has not been fully explored, its expression could provide an important mechanism for BK_Ca_ channel modulation and regulation of uterine contraction. Table 1 presents a summary of the known myometrial splice variants and their modified functions.

**Table 1:** **BK_Ca_ channel splice variants expressed in the myometrium**.

**Splice variant name**	**Affected domain**	**Number of amino acids added**	**Functional modification**	**References**
mK44	S0-S1 loop	44	decreased voltage and Ca^2+^ sensitivity, endoprotease cleavage	Korovkina et al., 2001, 2006; Curley et al., 2004
SV1	S1	33	endoplasmic reticulum retention	Zarei et al., 2001, 2004
STREX	S8-S9 loop	59	increased voltage and Ca^2+^ sensitivity, switches from PKA activation to inhibition	Saito et al., 1997; Benkusky et al., 2000; Tian et al., 2001; Zhu et al., 2005

### Trafficking

Membrane trafficking of the BK_Ca_ channel regulates a wide variety of physiological processes including pregnancy (Song et al., 1999), aging (Marijic et al., 2001), and aldosterone-induced K^+^ secretion from the gut (Sorensen et al., 2008). Two regions that control BK_Ca_ channel surface localization are the intracellular C-terminal linker between the RCK1 and RCK2 domains (Lee et al., 2009; Chen et al., 2010) and an actin-binding domain in the C terminus (Zou et al., 2008). In addition, isoforms containing different C-terminal sequences have distinct trafficking to the cell surface (Kim et al., 2007a; Ma et al., 2007).

Variation of the α-subunit by alternative splicing can add or delete signal sequences that modify channel localization by facilitating its retention in or targeting to intracellular organelles, including the ER (Zarei et al., 2001; Chen et al., 2010) and mitochondria (Singh et al., 2013). In rat myometrium, a splice variant containing the SV1 exon is retained in the ER, thereby preventing surface localization and affecting cell excitability (Zarei et al., 2001, 2004). In addition to splicing, co-expression with the auxiliary β1-subunit enhances internalization of the BK_Ca_ α-subunit into endosomes, thus controlling its membrane localization (Toro et al., 2006). Likewise, a related β4-subunit has an ER retention signal at its C terminus and prevents the α-subunit from exiting the ER (Shruti et al., 2012). As noted above, ER retention mechanisms have been explored in the myometrium, but their physiological relevance in modulating uterine contractility during pregnancy is still unknown.

### Mitochondrial localization

A mitochondrial BK_Ca_ (mitoBK_Ca_) channel was first identified by patch clamp studies performed on mitoplasts prepared from human glioma cells (Siemen et al., 1999). The structure of mitoBK_Ca_ is similar to the plasmalemmal BK_Ca_ except for the inclusion of a mitochondrial-targeting sequence, DEC, in the C-terminal region (Singh et al., 2013). Located in the inner mitochondrial membrane, mitoBK_Ca_ channels appear to be structurally and functionally coupled to the respiratory chain (Bednarczyk et al., 2013). In cardiac myocytes, activation of mitoBK_Ca_ channels attenuates mitochondrial Ca^2+^ overload (Sato et al., 2005). A similar effect is observed after activation of mitochondrial ATP-sensitive K^+^ channels, but these effects seem to be independent (Sato et al., 2005). The link between the mitoBK_Ca_ channel and myometrial function has not been explored. However, disruption of mitochondrial function decreases the amplitude and frequency of spontaneous contractions in non-pregnant mouse uterus, and some data suggest that this effect is, at least in part, mediated by Ca^2+^-activated K^+^ channels, such as the BK_Ca_ channel (Gravina et al., 2010). Notably, the effect occurs through modulation of Ca^2+^ influx and membrane potential. The idea that mitoBK_Ca_ functions in the myometrium is appealing. For example, activation of mitoBK_Ca_ improves mitochondrial respiratory function and thus protects the heart from ischemic injury (Xu et al., 2002). Moreover, mitoBK_Ca_ channels are more sensitive to hypoxia than plasma membrane BK_Ca_ channels in glioma cells (Gu et al., 2014), suggesting functional differences between these forms. Therefore, further work is required to determine (i) whether the mitochondria-dependent modulation of Ca^2+^ levels and uterine contractility changes during pregnancy, and (ii) whether mitoBK_Ca_ function affects mitochondria to accommodate changes in Ca^2+^ dynamics in the myometrium.

### Membrane compartmentation

Localization of proteins in cholesterol- and sphingolipid-rich membrane microdomains has been proposed as a mechanism to modulate membrane excitability and intracellular signaling (Razani et al., 2002). Several lines of evidence indicate that such microdomains play important roles in controlling myometrial excitability. First, the number of a specific type of microdomain, caveolae, increases in myometrial cells toward the end of pregnancy (Turi et al., 2001). Second, two isoforms of the scaffolding proteins that form caveolae, caveolin-1, and caveolin-2, are down regulated by estrogen (Turi et al., 2001) and labor (Chan et al., 2014). Third, depletion of membrane cholesterol and consequent disruption of membrane microdomains, induces an increase in uterine contractions and Ca^2+^ transients (Smith et al., 2005). Finally, multiple studies have shown that BK_Ca_ channels localize to membrane microdomains in both cells used for heterologous expression and smooth muscle cells (Bravo-Zehnder et al., 2000; Babiychuk et al., 2004). For example, co-localization of BK_Ca_ channels with downstream effectors and other receptors in caveolae alters channel function in vascular smooth muscle cells (Lu et al., 2010).

The discrete membrane localization of the BK_Ca_ channel with its effectors and regulators might be an important mechanism to modulate BK_Ca_ function in myometrium. In support of this idea, a sub-population of BK_Ca_ channels in MSMCs localizes to caveolae where they associate with both structural components of caveolae, caveolin-1, and caveolin-2, and cytoskeletal proteins, α- and γ-actin (Brainard et al., 2005). Specific down-regulation of caveolin-1 decreases BK_Ca_ currents and alters localization of BK_Ca_ channels from detergent-resistant to detergent-soluble membrane microdomains (Brainard et al., 2009). This effect is also observed by deleting the entire caveolin-binding motif in the C terminus of the BK_Ca_ channel (Alioua et al., 2008) or by mutating key amino acids in this region (Brainard et al., 2009). Moreover, disruption of caveolae by depletion of membrane cholesterol or depolymerization of the actin cytoskeleton increases BK_Ca_ activity in human MSMCs (Brainard et al., 2005). Conversely, cholesterol depletion decreases BK_Ca_ activity in rat MSMCs (Shmygol et al., 2007b). These contradictory observations might be explained if the cholesterol-depleting agent used in both studies differentially affected other membrane-bound proteins such as Ca^2+^ or K^+^ channels (Levitan et al., 2010). Nonetheless, it is tempting to speculate that differential localization of BK_Ca_ isoforms within caveolar domains of the plasma membrane partially explains the Ca^2+^-insensitive BK_Ca_ currents that are observed in laboring myometrium (Khan et al., 1993).

### Posttranslational modifications

The BK_Ca_ channel possesses numerous phosphorylation sites, and the phosphorylation state of these residues can regulate channel activity (Toro et al., 1998; Schubert and Nelson, 2001; Kyle et al., 2013). Below, we discuss three potential kinase modulators of BK_Ca_ channel activity in the myometrium: protein kinase A (PKA), protein kinase C (PKC), and protein kinase G (PKG).

In the myometrium, the association of PKA with the plasma membrane is regulated by progesterone and labor (Ku and Sanborn, 2002; Ku et al., 2005). Activation of the PKA pathway by cyclic AMP contributes to uterine quiescence during pregnancy through phosphorylation of various proteins (Lopez Bernal, 2007; Tyson et al., 2008). The BK_Ca_ channel is one such target; in non-pregnant myometrium, PKA inhibits BK_Ca_ channels, whereas in pregnant myometrium, phosphorylation by PKA activates the channel (Perez and Toro, 1994). This disparity may be explained by the fact that, as mentioned in section Splice variants, different splice variants of the BK_Ca_ channel respond in distinctive ways to PKA modulation (Tian et al., 2001; Zhou et al., 2001).

PKC is a serine/threonine kinase activated by increasing intracellular levels of diacylglycerol or Ca^2+^. In vascular SMCs, PKC directly phosphorylates the BK_Ca_ channel α-subunit, reducing its activity (Schubert and Nelson, 2001; Zhou et al., 2010). In these cells, PKC can also reduce BK_Ca_ channel activity indirectly by decreasing the release of Ca^2+^ sparks from the sarcoplasmic reticulum (Bonev et al., 1997; Hristov et al., 2014). Although the PKC modulation of agonist-dependent myometrial contractions has been explored (Phillippe, 1994; Breuiller-Fouche et al., 1998; Eude et al., 2000), the role of BK_Ca_ channels in this process remains elusive.

PKG, a serine/threonine-specific protein kinase that is activated by intracellular cyclic GMP, enhances BK_Ca_ activity by direct phosphorylation of serine residues (Alioua et al., 1998; Kyle et al., 2013). In SMCs, PKG has been shown to activate BK_Ca_ channels (Robertson et al., 1993; Archer et al., 1994; Zhou et al., 1996). Likewise, PKG enhances the activity of BK_Ca_ channels originally cloned from myometrium and subsequently expressed in a heterologous system (Zhou et al., 1998). Furthermore, PKG activation increases the activity of BK_Ca_ channels in myometrium (Zhou et al., 2000b), suggesting a role for PKG in maintaining uterine quiescence by modulation of BK_Ca_ channel activity. Functional contraction studies aimed at dissecting the effects of PKG on BK_Ca_ currents in non-pregnant and pregnant myometrium are required to elucidate whether this interaction has a role in the myometrium during pregnancy or labor.

## Extrinsic mechanisms of BK_Ca_ channel modulation

### Interaction with auxiliary proteins

The pore-forming BK_Ca_ channel α-subunits can associate with and be regulated by auxiliary β- and γ-subunits (Knaus et al., 1994b; Tanaka et al., 1997; Yan and Aldrich, 2012). Four distinct β-subunits proteins (β1-4) have been found to regulate the function and localization of the BK_Ca_ channel α-subunit (Knaus et al., 1994a; Wallner et al., 1999; Behrens et al., 2000; Brenner et al., 2000; Uebele et al., 2000). We will focus on the β1- and β2-subunits as these are expressed in MSMCs (Behrens et al., 2000; Chan et al., 2014). In addition, four members of a γ-subunit family, also known as leucine-rich repeat-containing (LRRC) proteins, that associate with the BK_Ca_ channel α-subunits: LRRC26 (γ1), LRRC52 (γ2), LRRC55 (γ3), and LRRC38 (γ4) (Yan and Aldrich, 2012) will be examined.

#### β-subunits

The β1-subunit is the predominant β-subunit in the myometrium. Association with β1 decreases the voltage dependency and enhances the apparent Ca^2+^-sensitivity of the BK_Ca_ channel α-subunits (McManus et al., 1995; Wallner et al., 1995; Tanaka et al., 1997; Lorca et al., 2014). The β1-subunit also modulates the membrane trafficking (Toro et al., 2006; Kim et al., 2007b), mobility (Yamamura et al., 2012), pharmacology (Giangiacomo et al., 2000), and alcohol and estrogen sensitivity (Valverde et al., 1999; Feinberg-Zadek and Treistman, 2007) of the α-subunits. In human myometrium, expression of both α- and β1-subunits decreases at the onset of labor (Matharoo-Ball et al., 2003; Gao et al., 2009; Chan et al., 2014). Their association with one another is not altered at this time (Matharoo-Ball et al., 2003), suggesting that dissociation of BK_Ca_ channels from accessory β1-subunits is not a mechanism to alter channel activity during pregnancy. However, certain variants of the BK_Ca_ channel α-subunit can be modulated differentially by the β1-subunit (Lorca et al., 2014), thus acting to fine tune the properties of BK_Ca_ to best fulfill its cell type-specific functions.

Similarly to β1, β2 increases BK_Ca_ channel Ca^2+^ and voltage sensitivity (Wallner et al., 1999), although the mechanisms of modulation may differ (Orio and Latorre, 2005; Yang et al., 2008; Lee et al., 2010). In addition to enhancing the activity of the α-subunit, the β2-subunit inactivates the channel currents by N-type inactivation (Wallner et al., 1999; Xia et al., 2003). Consistent with the idea that β2 inhibits uterine contractility during pregnancy, progesterone (which is high until the end of pregnancy) increases the expression of the BK_Ca_ α-subunit but decreases expression of β2 in MSMCs (Soloff et al., 2011).

#### γ-subunits

The γ1–γ4 subunits belong to a subgroup of the LRRC protein family, the “Elron” cluster, so named because they contain only the extracellular LRR region (Dolan et al., 2007). The effect of these auxiliary proteins on BK_Ca_ activity is remarkable, inducing shifts between −140 mV and −20 mV in the channel's voltage-activation curve in the absence of Ca^2+^ (Yan and Aldrich, 2012), thus providing strong modulation of channel function. In particular, the γ1-subunit enhances the voltage-dependency of BK_Ca_ channel activation, allowing activation at resting membrane potential and intracellular Ca^2+^ concentrations (Yan and Aldrich, 2010). This effect requires at least four γ1-subunits to associate with the pore forming α-subunits (Gonzalez-Perez et al., 2014). The γ1-subunit also reduces the sensitivity of the BK_Ca_ channel to its opener mallotoxin (Almassy and Begenisich, 2012). Likewise, the γ2-subunit has been shown to modulate a BK_Ca_-related pH-sensitive channel (Slo3) in sperm (Yang et al., 2011).

An extensive study by Yan and Aldrich (2012) showed that all four γ-subunits are expressed in the human uterus. This finding is intriguing because myometrial BK_Ca_ channel activity is significantly higher in women at labor than in non-pregnant women; in fact, at labor, BK_Ca_ activity is independent of intracellular Ca^2+^ (Khan et al., 1993). Thus, it is feasible that increased activity of the BK_Ca_ channel in labor is mediated by γ-subunit association. Further analysis of the biophysical properties of the myometrial BK_Ca_ channel at different gestational stages is necessary to elucidate its modulation by γ-subunits.

### Modulation by G-protein coupled receptors

#### Adrenergic modulation

Catecholamines, such as epinephrine and norepinephrine, have been well described to play a pivotal role in controlling uterine contraction through various G protein-coupled receptors (GPCRs), specifically the α- and β-adrenergic receptors (AR) (Bulbring and Tomita, 1987). Activation of α- and β-AR trigger two main signaling pathways: (i) activation of G_s_- or G_i_-protein, activation/inhibition of adenylyl cyclase (AC), and changes in cyclic AMP (cAMP) levels, and (ii) activation of G_q/11_-protein, production of inositol 1,4,5-triphosphate (IP_3_) and diacylglycerol (DAG), and an increase in intracellular Ca^2+^.

Clinically, β-AR agonists have been used as tocolytic agents, inducing relaxation of the myometrial smooth muscle through membrane hyperpolarization. However, the adverse cardiovascular and metabolic side effects in the mother and fetus (Jeyabalan and Caritis, 2002; Berkman et al., 2003) have dampened their effectiveness and limited their usage. Hence, a better understanding of the pathways downstream of adrenergic signaling might aid the design of new tocolytic agents. Interestingly, one of the main effectors of adrenergic signaling pathways involved in myometrial contractility is the BK_Ca_ channel.

In both the myometrium and lipid bilayers isolated from MSMCs, activation of β-AR increases Ca^2+^-activated K^+^ currents, which are likely mediated by BK_Ca_ channels (Toro et al., 1990; Anwer et al., 1992). Moreover, selective activation of β_2-AR_ increases AC activity, resulting in increased cAMP levels, activation of PKA, and increased BK_Ca_ currents (Zhou et al., 2000a). When both α_2_- and β_2_-AR are stimulated in MSMCs from a pregnant woman, a synergistic increase in BK_Ca_ current is observed, likely due to concomitant activation of AC by both Gβγ_*i*_-subunit and Gα_s_ (Zhou et al., 2000a). Two findings further support this observation: (i) β_2_-AR and the BK_Ca_ channel physically interact, and (ii) activation of β_2_-AR relaxes pregnant human myometrium, and this relaxation is attenuated by the BK_Ca_ channel blocker paxilline (Chanrachakul et al., 2004). Conversely, α_2_-AR stimulation antagonizes β_2_-AR in MSMCs from non-pregnant women. Therefore, a precise balance between α_2_- and β_2_-AR activity during pregnancy leads to increased BK_Ca_ channel function.

Interestingly, β_2_-AR and BK_Ca_ channels seem to be part of a macromolecule complex involving the A-kinase anchoring protein (AKAP79/150), PKA, and L-type Ca^2+^ channels (Liu et al., 2004), making the control of BK_Ca_ channel activity by phosphorylation and Ca^2+^ more efficient. Expression of AKAP79 and PKA are significantly lower in myometrial tissues from women in labor than in tissue from women not in labor (Ku et al., 2005). It has been proposed that these complexes are linked to caveolins and/or actin filaments (Lu et al., 2006), as observed for BK_Ca_ channel-angiotensin II signaling (Lu et al., 2010), and that disruption of these complexes and reduction of BK_Ca_ activity could lead to increased contractions at term.

Similar to the effects of β_2_-AR, selective stimulation of β_3_-AR activates single-channel and whole-cell BK_Ca_ currents in isolated human MSMCs (Doheny et al., 2005). Moreover, β_3_-AR activation inhibits both spontaneously occurring and oxytocin-induced contractions of myometrial strips from pregnant women, an effect that is abolished by blocking BK_Ca_ channels with iberiotoxin (Doheny et al., 2005). Hence, the adrenergic modulation of myometrial activity involves BK_Ca_ channel modulation and seems to vary according to the type of AR that is activated and the physiological state of the myometrium.

#### Modulation by other G-protein coupled receptors

The association of BK_Ca_ channels with, and their regulation by, GPCRs has been well established in other tissues. For example, M2 muscarinic receptors inhibit BK_Ca_ currents in tracheal SMCs (Zhou et al., 2008), whereas the G protein-coupled estrogen receptor 1 stimulates BK_Ca_ activity in coronary SMCs (Yu et al., 2011). Here we discuss five GPCRs that have been linked to uterine function: oxytocin, prostaglandin F_2α_, corticotropin-releasing hormone, nociceptin, and melatonin receptors.

The neuromodulator oxytocin increases the force and duration of myometrial contractions and is a widely used uterotonin to induce labor (Hawkins and Wing, 2012). The oxytocin receptor (OTR) is coupled to G_q/11_ protein and mediates both activation of the phospholipase C (PLC)/DAG/PKC pathway (Morrison et al., 1996) and IP_3_-induced intracellular Ca^2+^ increase (McKillen et al., 1999; Willets et al., 2009). OTR-dependent increases in intracellular Ca^2+^ lead to activation of BK_Ca_ channels (Zhou et al., 2007), which may serve as a negative feedback for oxytocin-induced uterine contractions. Further understanding of oxytocin's effects on BK_Ca_ channel activity will hopefully lead to strategies to avoid some of the side effects associated with the use of this labor-inducing drug.

Prostaglandins (PGs), derivatives from arachidonic acid, participate in several physiological processes, including regulation of smooth muscle contractility (Wong and Vanhoutte, 2010) and inflammation (Ricciotti and FitzGerald, 2011). The prostaglandin F_2α_ (PGF_2α_) is a potent uterotonin (Crankshaw and Dyal, 1994), and the levels of both PGF_2α_ and its receptor (FP) rise in the amniotic fluid at the onset of labor (Dray and Frydman, 1976; Brodt-Eppley and Myatt, 1999). Activation of the FP receptor, which is coupled to G_*q*_ protein, leads to increases in IP_3_, DAG, and intracellular Ca^2+^ levels. During labor, PGF_2α_ also regulates the expression of uterine contraction-associated proteins, such as connexin 43, OTR, and FP receptor, thus promoting uterine contractility (Xu et al., 2013). Inhibition of the FP receptor by the specific antagonist THG113 prevents pre-term labor in mouse (Peri et al., 2002) and induces marked relaxation of human myometrial tissue (Doheny et al., 2007). These effects may be explained by the fact that THG113 induces activation of BK_Ca_ channels in human MSMCs. However, the detailed mechanism of BK_Ca_ channel activation by this agent remains elusive (Doheny et al., 2007). Further studies will be necessary to determine the precise relationship between BK_Ca_ channel activity and signaling by PGF_2α_ or other PGs in the myometrium.

Corticotropin-releasing hormone (CRH), a polypeptide expressed in the placenta and uterus, activates the CRH receptors (CRH-R) expressed in the myometrium (Warren and Silverman, 1995). The plasma levels of CRH and its affinity for its receptors increase during pregnancy (Goland et al., 1986; Campbell et al., 1987; Hillhouse et al., 1993). CRH-R activation induces contraction of myometrium through different G-protein coupled signaling pathways, such as AC/cAMP/PKA and PLC/DAG/PKC (Grammatopoulos, 2007), an effect that appears specific to term pregnancy (Simpkin et al., 1999). CRH-Rs associate with the BK_Ca_ channel, and the two major subtypes, CRH-R1 and CRH-R2, regulate the expression of BK_Ca_ in MSMCs in a complicated manner (Xu et al., 2011). During pregnancy, CRH increases BK_Ca_ expression via CRH-R1, whereas it decreases BK_Ca_ expression via CRH-R2. Conversely, after onset of labor, CRH-R1 decreases BK_Ca_ expression, whereas CRH-R2 increases BK_Ca_ expression (Xu et al., 2011). These findings indicate that a finely tuned regulation of BK_Ca_ activity by CRH could control the transition of the myometrium from a quiescent to contractile state. How this occurs is yet to be fully defined.

Nociceptin is an opioid-related neuropeptide that is expressed in the uterus where it acts as a relaxant (Klukovits et al., 2010; Deak et al., 2013). The effect of nociceptin in myometrium is likely mediated by binding to its receptor, the orphan opioid receptor-like 1 (ORL-1), which is a G_i_ and G_s_ coupled receptor that regulates AC activity. In term pregnant rat uterus, activation of ORL-1 by nociceptin stimulates the production of cAMP (Klukovits et al., 2010). Interestingly, the relaxant effect of nociceptin is diminished by application of paxilline, a selective blocker of BK_Ca_ channels, suggesting that nociceptin-induced relaxation involves activation of BK_Ca_ channels (Klukovits et al., 2010).

Melatonin, a monoamine that regulates circadian rhythms, is expressed by pregnant human myometrium. In the myometrium, signaling via melatonin receptors-1 and -2 (MT1 and MT2) (Schlabritz-Loutsevitch et al., 2003) elicits several cellular signaling pathways, including inhibition of AC/cAMP formation and stimulation of Ca^2+^ transients through the PLC/IP_3_ pathway (Witt-Enderby et al., 2003). Melatonin increases BK_Ca_ channel activity in MSMCs in a PLC-dependent manner (Steffens et al., 2003), suggesting a role of melatonin in regulating myometrial excitability. However, melatonin can also enhance oxytocin-induced contraction of MSMCs (Sharkey et al., 2009). Both BK_Ca_ channels and melatonin are modulators of circadian rhythm behavior (Arendt and Skene, 2005; Meredith et al., 2006), which might impact the timing of parturition (Olcese et al., 2013), so additional evaluation of the effects of melatonin on BK_Ca_ channel activity and its role on uterine contractility might be necessary.

### Hormonal regulation

Numerous hormones regulate BK_Ca_ channel expression and activity in different tissues. Two relevant steroid hormones in the uterus, estrogens and progesterone, are key regulators for both maintaining uterine quiescence during pregnancy and for inducing labor at term. Although the levels of both hormones increase during pregnancy in humans (Boroditsky et al., 1978; Buster et al., 1979; Montelongo et al., 1992), changes in responsiveness of the target cells are key for their function. Here, we discuss ways in which BK_Ca_ might contribute to myometrial cell responsiveness to estrogens, progesterone, and also the hormone human chorionic gonadotropin.

The steroid hormone 17β-estradiol (E_2_) helps maintain pregnancy. As such, circulating E_2_ levels rise throughout pregnancy (Boroditsky et al., 1978; Buster et al., 1979; Montelongo et al., 1992), and the activity of the estrogen receptor α (ERα) is increased in myometrium near term (Mesiano and Welsh, 2007; Welsh et al., 2012). E_2_ regulates expression of the BK_Ca_ channel by species-specific mechanisms. For example, expression of the mouse BK_Ca_ gene (*mSlo1*) is up-regulated by E_2_ through activation of ERα and binding to estrogen response elements in the *mSlo1* promoter (Kundu et al., 2007). Expression of the human homolog (*KCNMA1* or *hSlo1*) is also up-regulated by E_2_ interaction with ERα, but through the phosphatidylinositol 3-kinase pathway (Danesh et al., 2011). Furthermore, E_2_ activation of ER decreases expression of the STREX variant in rat myometrium, mimicking the effect of pregnancy on this variant (Zhu et al., 2005). In addition, E_2_ augments the expression of the BK_Ca_ auxiliary β1-subunit in mouse uterus (Benkusky et al., 2002). Although less studied, the estrogen receptor β (ERβ) has also been suggested to play a role in myometrial quiescence and labor (Wu et al., 2000). Furthermore, ERβ is necessary for the E_2_-induced increase in BK_Ca_ currents in a neuronal cell line (Nishimura et al., 2008), but whether ERβ modulates myometrial BK_Ca_ currents has not been studied.

Although not yet fully explored, it is feasible that, at the onset of labor, E_2_ triggers activation of BK_Ca_ channel activity directly rather than by activation of ERα and up-regulation of BK_Ca_ gene expression in MSMCs. This is a strong possibility because BK_Ca_ channel expression is reduced at the end of pregnancy (Matharoo-Ball et al., 2003; Gao et al., 2009; Chan et al., 2014). Additionally, E_2_ can increase BK_Ca_ channel activity both in the presence (Valverde et al., 1999; De Wet et al., 2006) or absence (Wong et al., 2008) of the auxiliary β1-subunit by directly binding to the channel. An E_2_-dependent increase in BK_Ca_ channel activity has also been observed in uterine vascular SMCs (Hu et al., 2011). However, a lower concentration of E_2_ reduces BK_Ca_ currents and induces proteosomal degradation of the BK_Ca_ α-subunit (Korovkina et al., 2004). Hence, further studies are necessary to address the physiological significance of the E_2_-BK_Ca_ channel interaction in the myometrium.

Myometrial quiescence during pregnancy is, in part, attributable to high plasma levels of the steroid hormone progesterone. Progesterone acts through its receptor PR to inhibit expression of contraction-associated proteins such as OTR, connexin 43, and cyclooxygenase-2, a key enzyme in the biosynthesis of prostaglandins (Renthal et al., 2010; Williams et al., 2012). Progesterone has been shown to inhibit BK_Ca_ channel currents in human sperm (Mannowetz et al., 2013) as well as in heterologous expression systems (Wong et al., 2008), suggesting a direct interaction between PR and the BK_Ca_ α-subunit. However, other evidence indicates that progesterone regulates expression of BK_*Ca*._ For example, longer progesterone treatment increases mRNA and protein expression of the BK_Ca_ α-subunit in human immortalized MSMCs. Likewise, progesterone treatment decreases the expression of the β2-subunit (Soloff et al., 2011) without changing the expression of β1-subunit in mouse uterus (Xu et al., 2011). Although the effects of progesterone are wide and complex in the myometrium, elucidation of its effects on BK_Ca_ channel activity and expression will help to inform our understanding of the regulation of myometrial function by this hormone.

The human chorionic gonadotropin (hCG) is a glycoprotein produced mainly by the placenta. In addition to its role in sustaining early pregnancy, hCG may also participate in maintaining uterine quiescence during pregnancy. One study reported that hCG induces a potent relaxation of human myometrium *in vitro*, an effect partially attributable to an hCG-dependent increase in BK_Ca_ currents in MSMCs (Doheny et al., 2003). Simultaneously, another study found that certain unidentified chorionic-derived factors reduce oxytocin-mediated contraction in guinea pig myometrium in a paracrine manner, an effect that involves the activation of myometrial BK_Ca_ channels (Carvajal et al., 2003). Thus, BK_Ca_ channel seems to be a predominant effector of the uterorelaxant effects of chorionic-derived factors, including hCG.

### Other modulators

Other modulators of vascular smooth muscle such as nitric oxide (NO) and certain eicosanoids have been reported to change BK_Ca_ channel activity in the myometrium. NO is a gaseous molecule that acts as a potent vasodilator mainly via activation of soluble guanylyl cyclase and production of cGMP in smooth muscle. NO production increases during pregnancy (Choi et al., 2002), and decreases toward labor, suggesting a role in regulating uterine contractility. NO has been shown to increase the open probability of the BK_Ca_ channel in human MSMCs (Shimano et al., 2000), but whether this occurs by a direct interaction or by cGMP-dependent pathways is unknown.

Another modulator of BK_Ca_ channels in the myometrium is the non-prostanoid eicosanoid, 5,6-epoxyeicosatrienoic acid (5,6-EET), a metabolite of arachidonic acid. The 5,6-EET isomer, the most abundant eicosanoid isomer in myometrial tissue (Zhang et al., 2007), reduces oxytocin-induced contractions in human pregnant myometrium by increasing BK_Ca_ currents (Pearson et al., 2009). Additional studies should elucidate the nature of this interaction and its physiological significance in the myometrium, as well as in other tissues.

## Concluding remarks

During pregnancy, the myometrium must remain in a quiescent, relaxed state, and the MSMCs must remain hyperpolarized. At term, however, the MSMCs convert to a more depolarized state to allow the myometrium to become contractile. Modulation of BK_Ca_ channel function is pivotal for proper regulation of both these states. Thus, enhanced activity of BK_Ca_ channels might underlie myometrial quiescence during pregnancy. Conversely, reduced activity of this channel might result in earlier labor, and failure to properly modulate channel activity at the end of labor might interfere with the transition to a contractile state. Thus, it is perhaps not surprising that so many mechanisms function to regulate the BK_Ca_ channel and thus fine-tune the excitability of the myometrium. In addition to those regulators that are known to regulate BK_Ca_ in the myometrium, numerous modulators of BK_Ca_ channel activity have been described in different tissues and under different physio(patho)logical states. Complete understanding of these modulatory mechanisms will provide opportunities to develop precise treatments for labor mistiming and dysfunction.

### Conflict of interest statement

The authors declare that the research was conducted in the absence of any commercial or financial relationships that could be construed as a potential conflict of interest.
